# Prognostic Value of Preoperative Hemoglobin in Patients Undergoing Radical Prostatectomy for Localized Prostate Cancer

**DOI:** 10.3390/cancers17162633

**Published:** 2025-08-12

**Authors:** Dominik Enderlin, Lea Hertelendy, Josias Bastian Grogg, Franz von Stauffenberg, Daniel Eberli, Cédric Poyet

**Affiliations:** 1 Department of Urology, University Hospital of Zürich, 8091 Zürich, Switzerland; dominik.enderlin@usz.ch (D.E.);; 2 Department of Urology, Stadtspital Triemli, 8063 Zürich, Switzerland

**Keywords:** prostate cancer, preoperative, hemoglobin, progression-free survival, prostatectomy

## Abstract

**Background**: Hemoglobin (Hb) has been identified to be an independent prognostic marker for oncological outcomes in several malignancies. However, the impact of Hb levels before radical prostatectomy (RP) in localized prostate cancer remains unclear. **Methods**: Preoperative Hb levels were retrospectively collected from patients, who underwent RP from 2016 to 2022. Hb levels were analyzed as continuous and binary variables. For binary analysis, the cohort was divided into high-Hb (≥150 g/L) and low-Hb (<150 g/L) groups using the median as a cutoff. We used Spearman rank correlation to assess possible associations between Hb and continuous variables and logistic regression for Hb and binary variables. To assess the impact of preoperative Hb on recurrence-free survival (RFS), adjuvant treatment free survival (TFS), and metastasis-free survival (MFS), univariate and multivariate Cox regression analyses were performed. **Results**: A total of 567 patients were included in the analysis. Higher Hb levels, both when analyzed as a continuous variable and when divided in high and low groups, were inversely correlated with age (*p* < 0.001) and the International Society of Urological Pathology (ISUP) grade (*p* = 0.005 or *p* = 0.028, respectively). Patients in the high-Hb group showed a decreased risk of extraprostatic disease (≥pT3) (odds ratio [OR] 0.71, 95%-CI: 0.50–0.99, *p* = 0.047). In univariate cox regression analysis, high-Hb patients had a significantly longer RFS compared to the low-Hb group (hazard ratio [HR] 0.64, 95%-CI: 0.44–0.92, *p* = 0.015). When adjusting for age, ISUP grade, positive surgical margin, prostate specific antigen, nodal status, and ≥pT3, this effect was no longer statistically significant (HR 0.76, 95%-CI 0.56–1.22, *p* = 0.178). Hb was not a significant prognostic factor for TFS or MFS. **Conclusions**: In this large cohort, lower preoperative Hb values were associated with a more aggressive tumor grading and shorter RFS. However, we were unable to identify Hb as an independent predictor of oncological survival outcomes.

## Simple Summary

In several cancer types, lower hemoglobin levels are associated with impaired oncological survival outcomes. The effect of hemoglobin levels before curative surgery for patients with localized prostate cancer on patient survival remains unclear. The aim of this study was to assess the prognostic value of preoperative hemoglobin in prostate cancer patients undergoing radical prostatectomy. We found lower hemoglobin levels to be associated with a more aggressive tumor grading and shorter relapse time. However, hemoglobin did not remain an independent prognostic marker when assessed with other known clinico-pathological factors.

## 1. Introduction

Anemia is a common laboratory finding in daily clinical practice, affecting around 25% of the world’s population in 2021 [1,2]. This number has remained relatively stable over time, having only declined slightly from 28% in 1990 to its current level [2]. Preschool children and women are usually particularly affected by anemia [1,3]. There are a myriad of possible causes, with the most common being iron deficiency, followed by chronic disease, infections or haemoglobinopathies, and gastrointestinal and gynecological conditions [1,4]. Causes are known to vary with sex, age, and geographic location [1,3].

Reduced hemoglobin (Hb) values are also a common finding in cancer patients; anemia is estimated to be present in up to 80% of patients depending on cancer type and current cancer specific treatment [5,6,7,8,9]. Furthermore, a comprehensive literature review including 60 publications found anemia to be an independent unfavorable prognostic factor in several cancer patient populations. For example, the relative risk of death was increased by 19% in lung cancer patients, 75% in patients with head and neck cancer, 47% in prostate cancer patients, and 65% in lymphoma patients [10].

The exact mechanisms by which reduced Hb values affect oncological outcomes have been researched heavily in recent years. Despite significant progress in understanding how cancer influences anemia and how anemia-related tissue hypoxia affects cancer development, these mechanisms are still not completely understood [11,12,13].

Focusing on prostate cancer, several studies have linked reduced Hb values to a poorer prognosis in advanced metastatic disease [14,15,16]. In more detail, a study by Mulders et al. found anemia to be significantly associated with shorter overall survival (OS) in patients with newly diagnosed metastatic prostate cancer in univariate and multivariate analysis [14]. In a similar study by Mori et al., lower Hb was associated with shorter progression free survival (PFS), OS, and cancer specific survival (CSS) in hormone sensitive metastatic prostate cancer [17]. In a different study by Dai et al. investigating castration resistant metastatic prostate cancer, anemia was linked to a shorter PFS and OS [16]. Similarly, Emrich et al. included over 1000 patients with metastatic prostate cancer receiving various treatment regimens and found anemia to be an independent predictor of shorter OS [15].

In patients undergoing radiation treatment for prostate cancer, anemia was associated with shorter OS, but not CSS or distant relapse [18]. However in this study, Dunphy et al. included every patient who underwent radiation treatment of the prostate in their center, but did not specify the stage of the disease at which treatment was administered [18]. So far, no data exists on the impact of preoperative hemoglobin values on pathological or oncological outcomes in patients undergoing radical prostatectomy (RP) for localized prostate cancer.

In this study, we therefore aim to close the above-described gap by examining the possible impact of preoperative Hb values on oncological outcomes in patients undergoing RP for localized prostate cancer.

## 2. Materials and Methods

### 2.1. Patient Data Acquisition

Patients who underwent RP for localized prostate cancer at the University Hospital of Zürich(Zürich, Switzerland) and were enrolled in the Prostate Cancer Outcomes Cohort Study (ProCOC) [19] between January 2016 and December 2022 were included in this study. Data was retrospectively collected until January 2025. Anemia was defined as a Hb value of <134 g/L, as per the hospital laboratory’s definition. Recurrence free survival (RFS) was defined as the duration, measured in months, from the date of surgery until detection of biochemical recurrence (defined as a two-time measurement of prostate specific antigen (PSA) of >0.1 µg/L after achieving non detectable PSA-levels following RP). Patients with PSA persistence after RP were excluded from RFS analysis. Metastasis free survival (MFS) was defined as the duration, measured in months, from the date of surgery until detection of metastasis. Treatment-free survival (TFS) was defined as the duration, measured in months, from the date of surgery to the initiation of any adjuvant therapy specifically aimed at treating prostate cancer.

This study was approved by the Ethics Committee of the Canton of Zürich (Ref. Nr. StV KEK-ZH-Nr. 06/08).

### 2.2. Statistical Analysis

Statistical analysis was performed using Hb as a continuous variable and as a binary variable. When used as a binary variable, the median Hb value was used as a cutoff to divide the patients into a high-Hb (≥150 g/L) and low-Hb (<150 g/L) group. Additionally, the cohort was divided into anemic (defined as Hb < 134 g/L) and non-anemic (≥134 g/L) groups, as per the hospital laboratory’s definition of anemia.

First, we used the Shapiro–Wilk normality test to test for the normality distribution of continuous variables. Next, we used the Spearman rank correlation test to explore possible associations between Hb and continuous variables. For the analysis of potential associations between Hb and binary pathological outcomes, we conducted a logistic regression analysis.

Afterwards, we first performed univariate Cox regression analysis to identify individual variables associated with RFS, TFS, and MFS. Then multivariate analysis was conducted to evaluate a possible independent prognostic impact of Hb on RFS, MFS, and TFS. For RFS and TFS, we used a multivariate model adjusting for preoperative PSA levels, age, final International Society for Urologic Pathology (ISUP) Grade in the prostatectomy specimen, presence of extraprostatic disease (≥pT3), positive surgical margin (PSM), and positive nodal status (pN1). For MFS, the multivariate model chosen could only adjust for the strongest univariate predictors, PSM and pN1, because of the low rate of metastasis occurrence during follow up.

Kaplan–Meier analysis was used to plot RFS curves for patients stratified by high-Hb versus low-Hb groups. The log-rank test was employed to compare survival curves. In cases where the crossing of the survival curves was observed in the Kaplan–Meier plot, the proportional hazards assumption was formally tested using Schoenfeld residuals.

Only results with a *p*-value < 0.05 were considered statistically significant. Data was analyzed with the statistic program R (R-Core-Team, Vienna, Austria, Version 4.4.1).

## 3. Results

### 3.1. Patient Characteristics

A total of 567 patients were available for analysis. The median follow-up time was 57 months (Interquartile range [IQR]: 25–72 months). The median age was 65 years (IQR: 60–70 years). The median Hb was 150 g/L (IQR: 141.5–156 g/L). A total of 51 patients (9%) presented with anemia at the time of surgery.

Final histopathology revealed 6 patients (1.1%) with an ISUP Grade 1 tumor, 204 (36.0%) with ISUP Grade 2, 216 (38.1%) with ISUP Grade 3, 77 patients (13.6%) with ISUP Grade 4, and 64 (11.3%) patients with ISUP Grade 5.

Biochemical recurrence occurred in 122 patients (21.5%), while 73 patients (12.9%) experienced PSA-persistence after surgery. During the follow up time, 136 patients (24.0%) received adjuvant treatment, 41 patients (7.2%) developed metastatic disease, and 15 patients (2.6%) died with only one patient (0.2%) dying of prostate cancer (Table 1).

### 3.2. Association Analysis

After using the Shapiro–Wilk normality test to confirm that continuous variables were not normally distributed, we used the Spearman rank correlation analysis to evaluate a potential association between Hb levels and the continuous variables age, ISUP Grade, or PSA. For the binary variables ≥pT3, pN1, and PSM, logistic regression analysis was conducted.

Higher Hb levels, both when analyzed as a continuous variable and when divided in high-Hb or low-Hb groups, were inversely associated with age (*p* < 0.001) and ISUP Grade (*p* = 0.005 and *p* = 0.028, respectively). Additionally, patients within the high-Hb group had marginally lower rates of extraprostatic disease (Odds ratio [OR] 0.71, 95%-Confidence interval [95%-CI] 0.50–0.99, *p* = 0.047). This effect could not be observed when Hb was analyzed as a continuous variable. No significant association between Hb and PSA, pN1 or PSM could be detected (Table 2).

When dividing patients into anemic and non-anemic groups, no significant association with any of the above described variables could be found (Supplementary Table S1).

### 3.3. Uni- and Multivariate Cox Regression Analysis

Univariate and multivariate Cox regression analyses, along with Kaplan–Meier and concordance index evaluations, were conducted to assess the prognostic significance of Hb levels, both as a continuous and binary variable, on RFS, TFS, and MFS.

**Figure 1:**
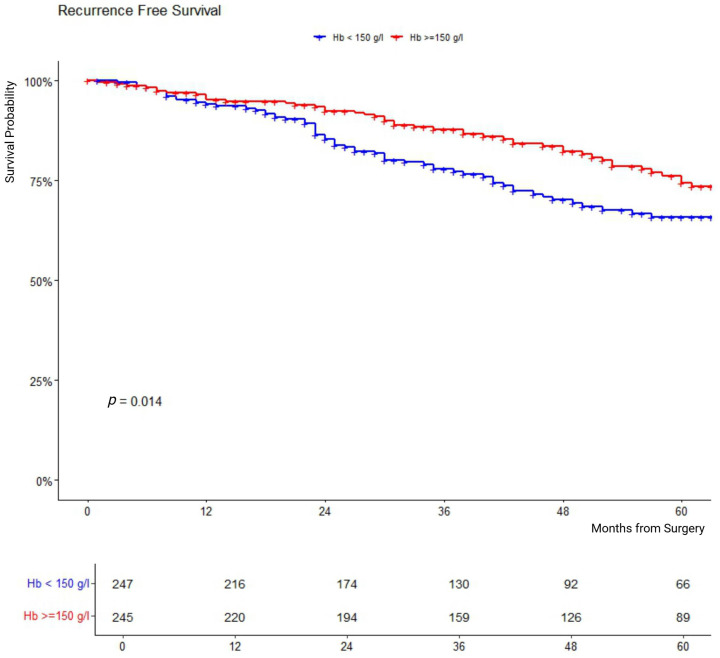
Kaplan–Meier analysis of RFS in months between high-Hb and low-Hb patient groups. Comparison of the Kaplan–Meier survival curves of the high-Hb (red) and low-Hb (blue) groups. *p*-values < 0.05 are considered statistically significant.

Univariate analysis revealed a significant association between high-Hb patients and longer RFS (Hazard ratio [HR] 0.64, 95%-CI 0.44–0.92, *p* = 0.015). This association was not present when Hb was used as a continuous variable. ≥pT3, PSM, pN1, ISUP Grade, and PSA were significantly associated (all *p* < 0.001) with shorter PFS (Table 3). In Kaplan–Meier analysis, patients in the high-Hb group had significantly longer RFS (*p* = 0.014) (Figure 1).

A visual inspection of the Kaplan–Meier Curves showed an early crossing of the survival curves, indicating a potential violation of the proportional hazards assumption of the used Cox model. Therefore, the proportional hazards assumption for the effect of high-Hb and low-Hb groups on RFS was formally tested using Schoenfeld residuals. We detected no significant violation of the assumption (Chi-square 1.39, 1 degree of freedom, *p* = 0.24), suggesting that the proportional hazards assumption can be considered valid over time. Given these results, we considered the Cox regression model reliable and appropriate to analyze the effect of high-Hb and low-HB groups on RFS.

Hb was not of prognostic significance in univariate analysis for TFS and MFS. On the other hand, ≥pT3, PSM, pN1, ISUP Grade, and PSA again were significantly associated (all *p* < 0.001) with shorter TFS and MFS (Supplementary Tables S2 and S3).

In multivariate analysis, preoperative Hb was not an independent predictor for a shorter RFS, TFS, or MFS (Table 4, Supplementary Tables S4 and S5), regardless of if analyzed as a continuous or binary variable. In addition, the addition of Hb did not significantly improve the Concordance Index of the multivariate model predicting PFS (Supplementary Table S6).

Finally, anemia was not associated with shorter RFS, TFS, or MFS in uni- or multivariate analysis (Supplementary Tables S2–S5, S7, and S8).

## 4. Discussion

To our knowledge, this is the first study to provide evidence that lower preoperative Hb is associated with a shorter RFS in patients undergoing radical prostatectomy, while not being an independent predictor.

In other solid malignancies, including cancers in the urogenital tract, low Hb or anemia has been shown to be of stronger prognostic relevance. For example, a meta-analysis by Huang et al. found preoperative anemia in patients with gastric cancer to be associated with shorter OS and disease-free survival (DFS) [6]. Similarly, Wilson et al. found preoperative anemia in patients with colorectal cancer to be linked to reduced DFS and OS as well [7]. Looking at urological malignancies, patients with anemia before nephroureterectomy for upper tract urothelial carcinoma (UTUC) had shorter RFS and CSS [20]. Similarly, patients with anemia before cystectomy for bladder cancer had shorter RFS and OS [21]. Comparable results have been produced for patients with other solid malignancies, including breast cancer, lung cancer, endometrial cancer, or ovarian carcinoma [5,22,23,24].

In prostate cancer, the role of Hb was investigated fairly extensively in metastatic disease, where reduced Hb levels were repeatedly associated with shorter PFS or OS [14,15,16]. An older study by Dunphy et al. found anemia before radiotherapy for prostate cancer to be an independent predictor of OS, but not CSS or MFS [18]. They investigated a cohort more comparable to ours than the studies focusing on metastatic disease. However, Dunphy et al. included all patients treated at their institution without declaring if the patients presented with localized or more advanced prostate cancer at the time of radiation. Nevertheless, their findings are partially similar to ours, as we also did not find a significant association between Hb and MFS. Contrary to our findings, Dunphy et al. observed an association between Hb and OS. The low number of deaths during follow-up in our cohort did not allow us to perform a similar analysis. Several studies have shown that patients who undergo surgery for localized prostate cancer tend to be relatively young and healthy. Conversely, patients who underwent radiation treatment for prostate cancer were older on average, had more comorbidities, and, additionally, a more advanced tumor stage [25,26,27]. When considering that anemia was linked to shorter OS only in people over the age of 60 in a general population cohort [28], the described link between anemia and reduced OS in patients undergoing radiotherapy is not unexpected.

In our study, we additionally found lower Hb levels before radical prostatectomy to be significantly associated with higher age, higher ISUP grade, and extracapsular disease. This mirrors findings in breast cancer or bladder cancer, where Hb was negatively correlated to higher T-stages [22,29]. Regarding tumor grade, similar results were produced for UTUC and colorectal cancer, where lower preoperative Hb values were also associated with a higher tumor grading [30,31]. Additionally, age has long been known to be inversely correlated with Hb in adult men [32].

The underlying causes of increased anemia rates in cancer patients have not been completely understood, although many mechanisms have been discussed in the literature. Cancer-associated anemia is thought to be partially mediated via an increase in inflammatory cytokines, like interleukin 6 or tumor necrosis factor alpha, that are believed to negatively interfere with iron metabolism [8,33]. Also, anemic cancer patients were shown to have lower erythropoietin (EPO) levels than expected, indicating a reduced EPO response [34]. Further possible causes include bone marrow infiltration and a generally poor nutritional status [9,35]. Additionally, specific deficiencies in vitamin B12, folic acid, and other vitamins have also been described [36,37]. Local bleeding caused by an invasive malignant disease can also lead to a relevant blood loss and result in anemia [38,39].

The possibility that anemia in cancer patients might also influence cancer progression has been discussed in the literature for several years [11,40,41,42]. A hypoxic tumor microenvironment can lead to increased angiogenesis, antiapoptotic signaling, and promote cancer progression and metastasis formation in prostate cancer [13,41,42]. In cervical and breast cancer, patient anemia was associated with a more hypoxic tumor microenvironment [11,12]. However, if this effect is also present in prostate cancer patients, in general, and whether this effect is relevant in our cohort with a low rate of anemic patients, in particular, remains unknown.

The outlined mechanisms suggest that a potential interplay between tumor hypoxia and tumor progression could provide an explanation for the observed association between lower hemoglobin levels and aggressive tumor biology.

Our results further imply that preoperative Hb is not an independent prognostic marker for survival outcomes in patients undergoing radical prostatectomy for localized prostate cancer. If we consider that many of the cancer-associated causes for anemia discussed above are indicators of systemic tumor manifestation, localized prostate cancer might not trigger such a pronounced effect in comparison to more advanced disease.

In our cohort, the median Hb was 150 g/L. Only 9% of patients presented with preoperative anemia, with around 41% of patients lying between 134 g/L and the median of 150 g/L. The reported median Hb value in our study is comparable to other cohorts explored for postoperative blood loss after RP, where the preoperative median Hb values were reported to be 146 g/L and 147 g/L [43,44]. Interestingly, the reported anemia rate in our study is considerably lower compared to other solid cancers, for example, the preoperative anemia rate was reported to be 36% in gastric cancer [6], 18–61% in colorectal cancers [7], and ca. 25% in breast and lung cancer [22,23]. Patients with localized prostate cancer undergoing radical prostatectomy are usually relatively healthy with at least a life expectancy of 10 years [45]. This aspect could be one of the reasons for the low rate of preoperative anemia observed in this cohort. Other cancer-associated causes for decreased Hb levels, like bone marrow infiltration or local bleeding, are not a relevant concern in the examined patient population with only localized prostate cancer.

Our study has some limitations, one being that Hb was only measured only once immediately before surgery. As Hb levels are known to fluctuate over time and even throughout the day [46,47], this could lead to a potential bias. However, given the large sample size and the use of Hb measurement methods that align with standard clinical practice, this approach is expected to yield results that are generalizable to patients undergoing radical prostatectomy. Further, due to the retrospective nature of our study, some potential confounders, for example, more details about nutritional status, potential vitamin B12 or folic acid deficiencies, or other potential factors influencing preoperative Hb [1,9,35] might not be accounted for. To address this limitation, future large prospective studies would be needed to better account for these potential cofounding factors and validate our findings under more controlled conditions. In our study, 9% of patients presented with anemia before RP, which could be considered a possible limitation. However, the reported median Hb value observed in this study is consistent with the reported values obtained in similar populations [43,44]. Moreover, given that our patient cohort largely consisted of otherwise healthy individuals [45], we cautiously propose that the observed Hb values may still be indicative of a general patient collective in a developed region.

## 5. Conclusions

In this large retrospective cohort, lower preoperative Hb values were associated with a more aggressive tumor grading and shorter RFS. However, we could not confirm Hb as an independent predictor of oncological survival outcomes. These results suggest that Hb could serve as a supplementary marker of overall tumor aggressiveness but should not currently alter management decisions. Further research could focus on the potential relationship between tumor-associated anemia and tumor–tissue hypoxia. Additionally, it could investigate if and how tissue hypoxia might contribute to tumor progression in localized prostate cancer.

## Figures and Tables

**Table 1:** Patient and tumor characteristics.

Variable	*n* or Median	% Or Interquartile Range
Number of patients	567	100
Age (y)	65	60–70
Preoperative hemoglobin (g/L)	150	141.5–156
PSA (µg/L)	7.64	5.05–12.08
Anemic patients (Hb < 134 g/L)	51	9.0
ISUP grade 1	6	1.1
ISUP grade 2	204	36.0
ISUP grade 3	216	38.1
ISUP grade 4	77	13.6
ISUP grade 5	64	11.3
≥pT3	209	36.8
PSM	70	12.3
pN1	78	13.8
Follow up time (m)	57	35–74
Biochemical recurrence	122	21.5
PSA-persistence	73	12.9
Any adjuvant therapy	136	24.0
Detection of metastasis	41	7.2
Death of any cause	15	2.6
Death caused by prostate cancer	1	0.2

Categorical values are presented with number (*n*) and corresponding percentage, while continuous variables are presented with median and interquartile range. PSA = prostate specific antigen; Hb = Hemoglobin; ISUP = International Society for Urologic Pathology; ≥pT3 = extraprostatic disease; PSM = positive surgical margin; pN1 = positive pathological nodal status.

**Table 2:** Association of hemoglobin values with clinico-pathological outcomes.

Preoperative Hemoglobin	Age		ISUP		PSA		≥pT3		pN1		PSM	
	SC	*p*-Value	SC	*p*-Value	SC	*p*-Value	OR (95%-CI)	*p*-Value	OR (95%-CI)	*p*-Value	OR (95%-CI)	*p*-Value
Hb (cont.)	−0.189	<0.001	−0.125	0.005	0.016	0.410	0.90 (0.97–1.00)	0.108	1.00 (0.98–1.02)	0.980	1.00 (0.98–1.01)	0.864
Hb ≥ 150 g/L vs. lower	−0.170	<0.001	−0.099	0.028	0.037	0.410	0.71 (0.50–0.99)	0.047	1.07 (0.68–1.74)	0.762	0.88 (0.61–1.27)	0.507

ISUP = International Society for Urologic Pathology; PSA = prostate specific antigen; ≥pT3 = extraprostatic disease; pN1 = positive pathological nodal status; PSM = positive surgical margin; SC = Spearman Coefficient; OR = odds ratio; 95%-CI = 95%-confidence interval; Hb = Hemoglobin; cont. = continuous.

**Table 3:** Univariate Cox regression analysis investigating different variables to predict recurrence free survival.

Variable	HR (95%-CI)	*p*-Value
Age (cont.)	1.03 (1.00–1.06)	0.030
≥pT3	1.95 (1.43–2.67)	<0.001
PSM	2.37 (1.64–3.43)	<0.001
pN1	4.21 (2.67–6.63)	<0.001
ISUP (cont.)	1.73 (1.45–2.06)	<0.001
PSA (cont.)	1.02 (1.01–1.03)	<0.001
Hb (cont.)	0.99 (0.97–1.00)	0.106
Hb ≥150 g/L vs. lower	0.64 (0.44–0.92)	0.015

pT3 = extraprostatic disease; PSM = positive surgical margin; pN1 = positive pathological nodal status; ISUP = International Society for Urologic Pathology; PSA = prostate specific antigen; HR = hazard ratio; 95%-CI = 95%-confidence interval; Hb = Hemoglobin; cont. = continuous.

**Table 4:** Multivariate Cox regression analysis investigating the value of hemoglobin to predict recurrence free survival adjusted for age, ISUP Grade, PSM, pN1, PSA, and ≥pT3.

Variable	HR (95%-CI)	*p*-Value
Hb (cont.)	0.99 (0.98–1.01)	0.501
Hb ≥ 150 g/L vs. lower	0.76 (0.56–1.22)	0.178

≥pT3 = extraprostatic disease; PSM = positive surgical margin; pN1 = positive pathological nodal status; ISUP = International Society for Urologic Pathology; PSA = prostate specific antigen; HR = hazard ratio; 95%-CI = 95%-confidence interval; Hb = Hemoglobin; cont. = continuous.

## Data Availability

The raw data supporting the conclusions of this article will be made available by the authors on request.

## Supplementary Materials

The following supporting information can be downloaded at: https://www.mdpi.com/article/10.3390/cancers17162633/s1, Figure S1: Hemoglobin Levels across ISUP Grades; Table S1: Univariable Cox Regression Analysis Investigating Different Variables to Predict Treatment Free Survival; Table S2: Univariable Cox Regression Analysis Investigating Different Variables to Predict Metastasis Free Survival; Table S3: Change in Concordance Index of a Multivariable Model Predicting Recurrence Free Survival Containing Age, ISUP Grade, PSM, pN1, PSA and ≥pT3 by Addition of Preoperative Hemoglobin Values; Table S4: Multivariable Cox Regression Analysis Investigating the Value of Hemoglobin to Predict Adjuvant Treatment Free Survival Adjusted for Age, ISUP Grade, PSM, pN1, PSA and ≥pT3; Table S5: Multivariable Cox Regression Analysis Investigating the Value of Hemoglobin to Predict Metastasis Free Survival Adjusted for PSM and pN1; Table S6: Change in Concordance Index of a Multivariate Model Predicting Recurrence Free Survival Containing Age, ISUP Grade, PSM, pN1, PSA and ≥pT3 by Addition of Preoperative Hemoglobin Values; Table S7: Univariate Cox Regression Analysis Investigating Anaemic Hb Values to Predict Recurrence Free Survival; Table S8: Multivariate Cox Regression Analysis Investigating the Investigating Anaemic Hb Values to Predict Recurrence Free Survival adjusted for Age, ISUP Grade, PSM, PSA, pN1 and ≥pT3.

## Abbreviations

The following abbreviations are used in this manuscript:

Hb	Hemoglobin
RP	Radical Prostatectomy
RFS	Recurrence Free Survival
TFS	Adjuvant Treatment Free Survival
MFS	Metastasis Free Survival
ISUP	International Society for Urological Pathology
≥pT3	Extraprostatic Disease
OR	Odds Ratio
HR	Hazard Ratio
IQR	Interquartile Range
95%-CI	95% Confidence Interval
PSM	Positive Surgical Margin
PSA	Prostate Specific Antigen
pN1	Positive Pathological Nodal Status
OS	Overall Survival
CSS	Cancer Specific Survival
DFS	Disease Free Survival
SC	Spearman Coefficient
UTUC	Upper Tract Urothelial Carcinoma
EPO	Erythropoietin
