# Supplementary material for: Prognostic Value of Preoperative Hemoglobin in Patients Undergoing Radical Prostatectomy for Localized Prostate Cancer
Source: Cancers (Basel). 2025 Aug 12;17(16):2633. doi: 10.3390/cancers17162633 (PMC12384518; doi:10.3390/cancers17162633)
Supplement: Supplementary file 1 [file cancers-17-02633-s001.zip › cancers-3784192-supplementary.pdf]

Supplementary Table S1: Association of anaemic Hb values with age, PSA and pathological outcomes

Variable	Age		ISUP		PSA		≥pT3		pN1		PSM	
	SC	p-value	SC	p-value	SC	p-value	OR (95%-CI)	p-value	OR (95%-CI)	p-value	OR (95%-CI)	p-value
Hb ≥134 g/l vs lower	-0.058	0.201	0.026	0.561	- 0.038	0.402	1.09 (0.61 – 2.03)	0.772	1.21 (0.54 – 3.27)	0.666	1.50 (0.78 – 3.15)	0.246

ISUP = International Society for Urologic Pathology, ≥pT3 = extraprostatic disease, pN1 = positive pathological nodal status, PSM = positive surgical margin, PSA = prostate specific antigen, OR = odds ratio, 95%-CI = 95%-confidence interval, Hb = Hemoglobin.

Supplementary Table S2: Univariate Cox Regression Analysis Investigating Different Variables to Predict Treatment Free Survival

**Variable**	**HR (95%-CI)**	**p-value**
Age (cont.)	0.99 (0.98 – 1.01)	0.102
≥pT3	3.87 (2.71 – 5.51)	**<0.001**
PSM	3.30 (2.35 – 4.81)	**<0.001**
pN1	8.75 (6.19 – 12.37)	**<0.001**
ISUP (cont.)	2.45 (1.92 – 2.63)	**<0.001**
PSA (cont.)	1.02 (1.01 – 1.03)	**<0.001**
Hb continuous	1.00 (0.98 - 1.01)	0.602
Hb ≥150 g/l vs lower	0.96 (0.78 – 1.33)	0.775
Hb ≥134 g/l vs lower	1.18 (0.62 – 2.25)	0.613

≥pT3 = extraprostatic disease, PSM = positive surgical margin, pN1 = positive pathological nodal status, ISUP = International Society for Urologic Pathology, PSA = prostate specific antigen, HR = hazard ratio, 95%-CI = 95%-confidence interval, Hb = Hemoglobin, cont. = continuous.

Supplementary Table S3: Univariate Cox Regression Analysis Investigating Different Variables to Predict Metastasis Free Survival

**Variable**	**HR (95%-CI)**	**p-value**
Age (cont.)	1.02 (0.92 – 1.07)	0.360
≥pT3	2.71 (1.80 – 4.10)	**<0.001**
PSM	3.89 (2.07 – 7.38)	**<0.001**
pN1	3.49 (2.33 – 5.23)	**<0.001**
ISUP	2.60 (1.82 – 3.51)	**<0.001**
PSA (cont.)	1.01 (1.01 – 1.01)	**<0.001**
Hb (cont.)	1.02 (0.99 – 1.05)	0.197
Hb ≥150 g/l vs lower	1.50 (0.79 – 2.84)	0.216
Hb ≥134 g/l vs lower	1.69 (0.41 – 7.01)	0.469

≥pT3 = extraprostatic disease, PSM = positive surgical margin, pN1 = positive pathological nodal status, ISUP = International Society for Urologic Pathology, PSA = prostate specific antigen, HR = hazard ratio, 95%-CI = 95%-confidence interval, Hb = Hemoglobin, cont. = continuous.

Supplementary Table S4: Multivariate Cox Regression Analysis Investigating the Value of Hemoglobin to Predict Adjuvant Treatment Free Survival Adjusted for Age, ISUP Grade, PSM, pN1, PSA and ≥pT3

**Variable**	**HR (95%-CI)**	**p-value**
Hb (cont.)	1.01 (0.99 – 1.03)	0.257
Hb ≥150 g/l vs lower	1.44 (0.98 – 2.13)	0.064
Hb ≥134 g/l vs lower	1.02 (0.53 – 1.96)	0.953

≥pT3 = extraprostatic disease, PSM = positive surgical margin, pN1 = positive pathological nodal status, ISUP = International Society for Urologic Pathology, PSA = prostate specific antigen, HR = hazard ratio, 95%-CI = 95%-confidence interval, Hb = Hemoglobin, cont. = continuous.

Supplementary Table S5: Multivariate Cox Regression Analysis Investigating the Value of Hemoglobin to Predict Metastasis Free Survival Adjusted for PSM and pN1

Variable	HR (95%-CI)	p-value
Hb (cont.)	1.02 (0.99 – 1.06)	0.111
Hb ≥150 g/l vs lower	1.63 (0.86 – 3.09)	0.138
Hb ≥134 g/l vs lower	1.40 (0.34 – 5.81)	0.645

PSM = positive surgical margin, pN1 = positive pathological nodal status, HR = hazard ratio, 95%-CI = 95%-confidence interval, Hb = Hemoglobin, cont. = continuous.

Supplementary Table S6: Change in Concordance Index of a Multivariate Model Predicting Recurrence Free Survival Containing Age, ISUP Grade, PSM, pN1, PSA and ≥pT3 by Addition of Preoperative Hemoglobin Values

Variable	Ci without Hb	Ci with Hb	p-value
Hb (cont.)	0.700	0.702	0.504
Hb ≥150 g/l vs lower	0.700	0.701	0.176

≥pT3 = extraprostatic disease, PSM = positive surgical margin, pN1 = positive pathological nodal status, ISUP = International Society for Urologic Pathology, PSA = prostate specific antigen, Ci = Concordance Index, Hb = Hemoglobin, cont. = continuous.

Supplementary Table S7: Univariate Cox Regression Analysis Investigating Anaemic Hb Values to Predict Recurrence Free Survival

Variable	HR (95%-CI)	p-value
Hb ≥134 g/l vs lower	1.47 (0.71 – 3.02)	0.299

HR = hazard ratio, 95%-CI = 95%-confidence interval, Hb = Hemoglobin.

Supplementary Table S8: Multivariate Cox Regression Analysis Investigating the Investigating Anaemic Hb Values to Predict Recurrence Free Survival adjusted for Age, ISUP Grade, PSM, PSA, pN1 and ≥pT3

Variable	HR (95%-CI)	p-value
Hb ≥134 g/l vs lower	1.41 (0.68 – 2.93)	0.355

≥pT3 = extraprostatic disease, PSM = positive surgical margin, pN1 = positive pathological nodal status, ISUP = International Society for Urologic Pathology, PSA = prostate specific antigen, HR = hazard ratio, 95%-CI = 95%-confidence interval, Hb = Hemoglobin.

Supplementary Figure S1: Haemoglobin Levels across ISUP Grades

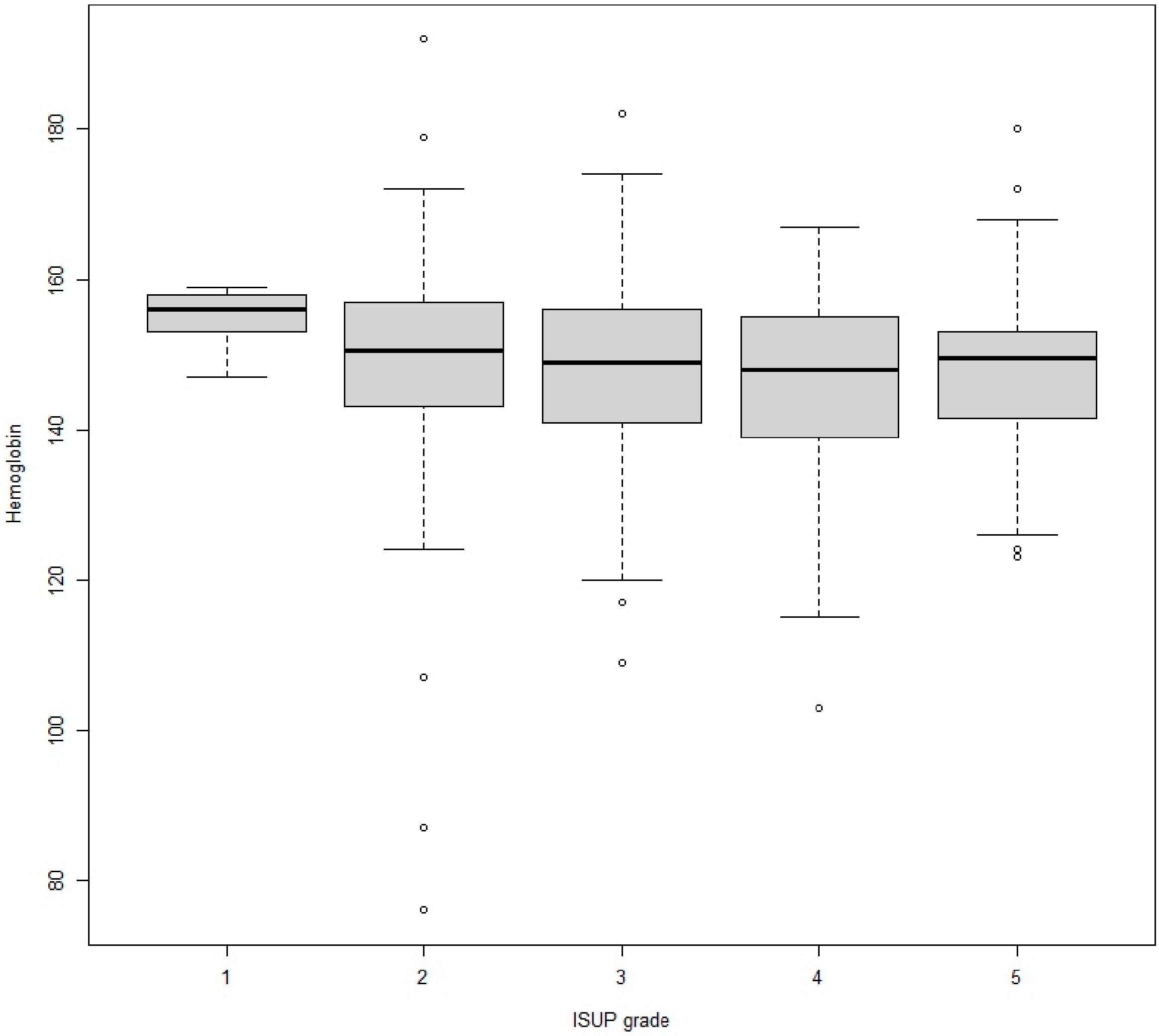

Depiction of the median Hemoglobin Levels in g/l with 95%-Confidence-Intervals across different International Society of Urological Pathology (ISUP) Grades.
